# A Customer Feedback Platform for Vehicle Manufacturing Compliant with Industry 4.0 Vision

**DOI:** 10.3390/s18103298

**Published:** 2018-10-01

**Authors:** Marianne Silva, Elton Vieira, Gabriel Signoretti, Ivanovitch Silva, Diego Silva, Paolo Ferrari

**Affiliations:** 1 Postgraduate Program in Electrical and Computer Engineering, Federal University of Rio Grande do Norte, Natal 59078-970, Rio Grande do Norte, Brazil; 2 Digital Metropolis Institute, Federal University of Rio Grande do Norte, Natal 59078-970, Rio Grande do Norte, Brazil; eltonviana@ufrn.edu.br; 3 Department of Computer Engineering and Automation, Federal University of Rio Grande do Norte, Natal 59078-970, Rio Grande do Norte, Brazil; gabrielsig@ufrn.edu.br; 4 School of Science and Technology, Federal University of Rio Grande do Norte, Natal 59078-970, Rio Grande do Norte, Brazil; diego@ect.ufrn.br; 5 Department of Information Engineering, University of Brescia, 25123 Brescia, Italy; paolo.ferrari@unibs.it

**Keywords:** Internet of Things, Internet of Intelligent Vehicles, Internet of Industrial Things, Industry 4.0, OBD-II, monitoring

## Abstract

In the last decade, the growth of the automotive market with the aid of technologies has been notable for the economic, automotive and technological sectors. Alongside this growing recognition, the so called Internet of Intelligent Vehicles (IoIV) emerges as an evolution of the Internet of Things (IoT) applied to the automotive sector. Closely related to IoIV, emerges the concept of Industrial Internet of Things (IIoT), which is the current revolution seen in industrial automation. IIoT, in its turn, relates to the concept of Industry 4.0, that is used to represent the current Industrial Revolution. This revolution, however, involves different areas: from manufacturing to healthcare. The Industry 4.0 can create value during the entire product lifecycle, promoting customer feedback, that is, having information about the product history throughout it is life. In this way, the automatic communication between vehicle and factory was facilitated, allowing the accomplishment of different analysis regarding vehicles, such as the identification of a behavioral pattern through historical driver usage, fuel consumption, maintenance indicators, so on. Thus, allowing the prevention of critical issues and undesired behaviors, since the automakers lose contact with the vehicle after the purchase. Therefore, this paper aims to propose a customer feedback platform for vehicle manufacturing in Industry 4.0 context, capable of collecting and analyzing, through an OBD-II (On-Board Diagnostics) scanner, the sensors available by vehicles, with the purpose of assisting in the management, prevention, and mitigation of different vehicular problems. An intercontinental evaluation conducted between Brazil and Italy locations shown the feasibility of platform and the potential to use in order to improve the vehicle manufacturing process.

## 1. Introduction

Vehicles manufactured today have several embedded sensors that are able to monitor numerous vehicle characteristics, such as: speed, engine temperature, brake operation, and a series of other information. Most of these vehicles can be connected with various types of devices (sensors, telephones, cameras), wireless communication protocols and broadcast media [1,2].

In this context, it is seen that the raw data from these devices and vehicle systems can be extracted by the vehicle itself in an automated way. All data can be exploited for interactions in a new context of processing and communication, revolutionizing the way vehicles are used. In fact, such interactions occur in the perspective of the Internet of Things (IoT), which enables the exchange of information between objects and platforms [3,4,5], promoting innovative business models and new user experiences through strong connectivity [6,7]. This emerging paradigm encompasses an infrastructure of hardware, software, and services that enables the interconnection between large volumes of distributed and heterogeneous smart things. As well as allowing them to communicate seamlessly with the user [5,8,9,10].

It is known that IoT is one of the most common and widely used technologies for the formation of smart environments based on sensors [11]. Currently, IoT is being implemented in a variety of scenarios, including environmental and urban monitoring, production, manufacturing, healthcare, home automation, transportation, and many others. The key idea behind IoT is the machine to machine communication in which each and every device is connected using sensor technology [11,12].

This scenario allows for the definition of an Internet of Intelligent Vehicles (IoIV) that integrates many important features of the IoT [12,13]. This technology intends to offer a series of new services, such as: fleet management systems, improvement of vehicle safety, efficient energy/fuel consumption, traffic planning, accident reduction, and, on another technological front, an important tool for the operation of autonomous vehicles [14,15]. The vehicle communication in IoIV is being derived through Vehicular Ad-hoc Networks (VANETs) [16,17], which are mobile networks for communication between cars and the external environment [18,19]. In essence, using this approach, the remote IoT control servers can monitor any of the moving vehicles in real time, enabling tracking to be done on a multitude of parameters [11].

Closely related to IoIV, emerges the concept of Industrial Internet of Things (IIoT), which is the current revolution seen in industrial automation [20,21]. IIoT, in its turn, relates to the concept of Industry 4.0, a term introduced in 2011 as an initiative of the German government with a focus on improving efficiency in the manufacturing industry [22,23]. Everything is getting smarter and the data generated at all levels of the production process are used to improve product quality, flexibility, and productivity [20,23].

This new industrial revolution has three main elements: the product and production network, the lifecycle of the product and, lastly, cyber-physical systems. First, the company’s production network, which integrates Manufacturing Execution Systems (MES) and Enterprise Resource Planning (ERP) systems, increases the level of automation at the plant. Thus, enabling the full exchange of real-time information for management purposes. The second element would be the information gathering throughout the product and production life cycles [24,25]. Finally, the Cyber-physical systems (CPS) are the integration between virtual and physical worlds. With the help of sensors and actuators, the software is integrated into all parts of the process, allowing a rapid information exchange, high process flexibility and precise control of the production process [24,26,27].

From a communications perspective, IIoT and CPSs rely largely on mobile Internet, i.e., telecommunication networks [28]. All practically relevant wireless networks in use today build on standards devised for computer networks or Wireless Personal Area Networks (WPANs). The upper layers are, in many cases, consistent with wired networks to retain compatibility, and the main challenge is again to ensure real-time and reliability capabilities [29,30]. Since the idea that everything in automation is connected and, e.g., that individual products or workpieces are parts of this ecosystem [28].

**Figure 1:**
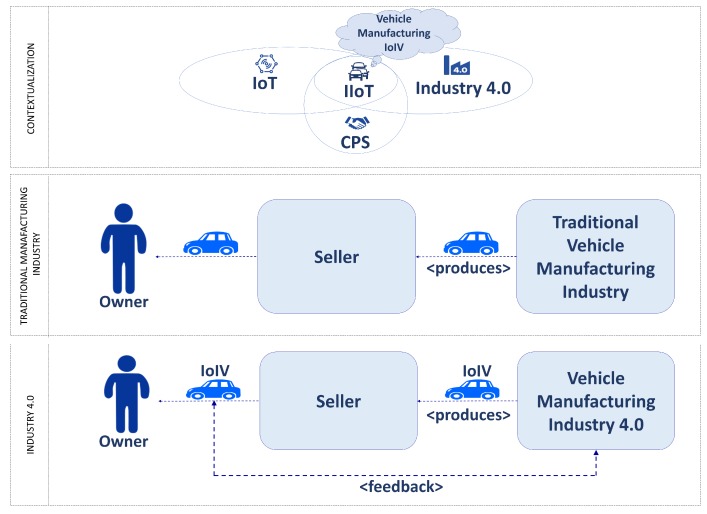
Customer Feedback in an IoIV and Industry 4.0 scenario.

Thus, it is realized that Industry 4.0 and Smart Cities have everything to do with customer feedback, therefore allowing for a more effective, precise and predictive decision-making process [31]. It also to Industry 4.0 aims to develop intelligent products, which collect and store information through their life cycle in an inherent way, giving feedback to the development and production [24,32]. Smart Cities, for their part, benefit to change the management of the city from a reactive to proactive administration [33]. Together, IoIV and Industry 4.0 have the potential to create a new paradigm for vehicle manufacturing organizations [34], enabling real-time customer feedback between the end-users (owners of vehicles) and factories. As a result, it will be possible to know if it is necessary to make a modification to a given series of products and what adjustments would be required in order to satisfy the customer needs in a more timely fashion. This feedback loop is shown in Figure 1.

For this purpose, since products that support IoIV are less developed at the moment [12], this article aims to perform an experiment with the customer feedback platform for Intelligent Vehicles in an Industry 4.0 perspective. An off-the-shelf OBD-II (On-Board Diagnostics) reader device that supports a Bluetooth connection is used to collect vehicle data directly from the ECU (Engine Control Unit) in real time. Using a smartphone with a Wi-Fi or mobile internet connection as the gateway for sending data to a server in the cloud. At the cloud, the platform is capable of collecting and analyzing raw data to detect and identify the occurrence of faults in the vehicles. Additionally, the platform implements an API (Application Programming Interface) supporting the following features:

(a) To guarantee compatibility with the entire range of OBD-II commands;
(b) A personalized holistic decoding of supported OBD-II commands according to specific vehicles;
(c) Enable parallel execution of OBD-II commands;
(d) Intelligent module for sending data based on unique communication technologies;
(e) Module for fog computing in order to pre-process the raw data;
(f) A transparent sending mechanism for visualization and analysis in the cloud;
(g) State machine to minimize the energy consumption of GPS (Global Positioning System) and communication protocols.

The remainder of this paper is organized as follows: Section 2 details some of the most relevant research works on the literature. Section 3 describes the proposed platform. In Section 4 we detail the evaluation of our approach. The results were obtained in an intercontinental evaluation performed in Brazil and Italy, as well as with different vehicles manufactures as described on Section 5. Finally, Section 6 concludes the paper and recommends directions for future studies.

## 2. Related Works

Automotive systems began to gain momentum in the same period of time as the California Air Resources Management Committee (CARB) and the Environmental Protection Agency (EPA) established resolutions to control emissions of gaseous pollutants. These resolutions propelled the creation of a system capable of operating self-diagnosis and alert the driver to possible defects in electronic components or in the emission control systems of the vehicle [35]. The system was called OBD-II and its first version was launched in 1988, when it became mandatory in all vehicles that would circulate in the state of California from that year forward [36]. Since 1996, all vehicles manufactured and marketed in the United States are required to have the OBD-II system. The European Union adopted a similar measure in 2003 while Brazil, Russia, and China followed the trend in 2010 [35].

For this reason, the researches with the use of OBD-II began with the purpose of emission control, as is the case of the works [35,37,38,39]. In [37], technical solutions for exhaust emissions are presented under real operating conditions of a vehicle. The author describes a road exhaust emissions research methodology with the use of information about the air flow that is supplied for the engine and the measured volumetric shares of particular fumes components (exhaust gas analyzer). As a result, it is possible to observe the inadequacy of the type-approval tests performed by manufacturers, used to ensure compliance with the emission standards, which differs from the real operating conditions of the vehicle.

Similarly, ref. [39] has developed and analyzed a model for measuring vehicle exhaust emissions. Five gases (CO, HC (SH_4_), NO_x_, CO_2_ and SO_2_) were analyzed while the vehicle was in normal operation. Using the OBD-II, several parameters could be used, like: vehicle speed, engine speed, fuel consumption, angular position of the throttle valve, cooling water, oil and exhaust gas temperature. The analysis indicates that the average emission rates (kg/h) for CO are more than 10 times higher than NO at idle speeds.

In like manner, ref. [35] has developed a platform capable of estimating the amount of carbon dioxide from existing vehicle sensor readings through the OBD-II. As a result, it has been found that the amount of CO_2_ content is directly linked to the combustion efficiency of the engine, i.e., if a vehicle releases very high amounts of the gas, it can be an indication that the vehicle has an electrical or mechanical problem, or even an indication of the quality of the fuel used.

On the other hand, ref. [38] has proposed a fuel consumption sensing and control system for vehicles (EcoDrive), implemented in an embedded platform, to improve fuel efficiency and reduce carbon emissions. The EcoDrive detects vehicle dynamics through the OBD-II and models various forces acting on the vehicle, i.e., propulsion, drivetrain loss, wind resistance and grade resistance, as functions of instant fuel consumption. In this way, the EcoDrive achieved an average of 20% higher fuel efficiency in urban road segments and 30% higher fuel efficiency on highways.

However, in more recent researches, the potential of the OBD-II to extract data from vehicles and generate useful information that can be used in various vehicular segments is beginning to be more broadly explored. Examples of this tendency are discussed in [40,41,42,43,44,45].

The work of [40], is focused on the development of a fleet management system using the OBD-II. The system aims to measure speed, distance, and fuel consumption of vehicles for tracking and computational analysis purposes. The data from the OBD-II is transmitted via Wi-Fi to a remote server. Then a database management system is implemented on the server to store the data, allowing different analyzes to be made.

In [41] the authors implemented a mobile diagnosing system that provides interfaces for the user. More precisely, estimating and diagnosing engine conditions through communications with the self-developed ECU. For the implemented system, a new protocol based on the OBD-II standard was designed and applied to receive engine data values of the developed ECU. The protocol is able to transmit 31 types of engine condition information simultaneously and sends the diagnostic trouble code. As a result, it is expected to provide user-centered diagnostic services and to avoid accidents caused by lack of proper car maintenance. Similarly, ref. [42] has presented a diagnostic system for hybrid electric vehicles and a data monitor. It was accomplished by using the OBD-II diagnostic system and an Android smartphone, through 3G mobile network, GPS and Bluetooth technology.

The authors [43] propose a smart vehicle telematics platform, connecting the OBD-II to the device and then to an application on a smartphone. It can be used to improve safety on the road, since it is capable of monitoring the vehicle information in real time. As a result, the platform is expected to help reduce the rate of serious accidents efficiently. In addition, the proposed system also provides geographic coordinates which, in turn, can help recover lost vehicles.

Equally important, ref [44] presented an experimental study of a Smart Tachograph system used to verify if speed limits and compulsory rest periods are been respected by drivers. As well as all the other works cited, they use the OBD-II for collecting the necessary data. It was found that the metrics selected for the tests are susceptible to data gaps, latency between Global Navigation Satellite System and odometry data and simplistic manipulations such as data scaling. This makes it more difficult to implement a fraud resistant system in comparison to the current version of the digital tachograph.

In addition to these works, ref. [45] stands out, since worldwide researches on the battery failure diagnostic system have not achieved remarkable accomplishments [46]. Thus, [45], conducted an experiment to examine the battery diagnosis in electric vehicles with the use of OBD-II. They found that, with the battery diagnosis system, it is possible to predict faults as well as to ensure the reliable operation of electric vehicles.

Furthermore, other significant contributions are emerging that encompass the theory and practice of Industry 4.0. After a literature review, it was noticed that few authors have specifically dealt with Industry 4.0 and IoIV focused on customer feedback. From those, the research from [47] stands out. In this work, the authors propose a non conventional approach for Industry 4.0 in order to manage a large amount of data generated by the sensors both inside and around the vehicle in the urban environment. Some benefits were recognized through the use of geo-localization information of the vehicles, discovering in real-time queue of events. This is useful for helping agencies with potential interest, like city planners and traffic agencies.

Similarly, ref. [48] proposed the use of autonomous connected vehicles for handling products between workstations in the manufacturing plant automatically. The results confirm their effectiveness in a typical production process.

Complementing, ref. [49] have proposed an approach for updating electronic brake control systems through mobile communication channels, making Software Updates Over The Air (SOTA) possible. Thus, it is expected to reduce expenses that vehicle manufacturers are facing when a software repair is needed, since with SOTA drivers themselves can carry out the update process.

Finally, on Table 1 a comparative summary between the characteristics of the works mentioned in this section is presented. There, it is possible to visualize their main similarities and differences. Each row represents one particular work and each of the four columns indicates topics (or features) and how they were covered in the given research. The for features analyzed are: Observed Sensors, Data Storage, Industry 4.0 and Mobile Applications.

The 13 works presented in Table 1 demonstrate distinct characteristics, and none contemplates all the four predetermined topic. In fact, it can be seen in the literature (through the The Observed Sensors feature) that the studies still do not use vehicle sensors in a holistic way as the proposed platform. Instead, they carry out analysis with some sensors in isolation. Likewise, the Data Storage feature shows that only a few studies have used online databases for future analysis, such as the proposed platform. With relation the Industry 4.0 feature, just the works [47,48,49] have addressed the topic, and none of those used the OBD-II for data collection. Finally, the last feature used for comparison was the use of an Mobile Applications, and it can be seen that only [35,38,41,42,43,44] have implemented a solution.

In this way, the relevance and the relationship of each one of the presented works with the proposed work are highlighted. Thus, from the discussion presented previously, it is clear that there are still gaps to be explored in this area, fostering the development of new solutions through OBD-II, IoIV and Industry 4.0 technologies. Within this context we have developed this work, which comes with the experiment of a customer feedback platform for Industry 4.0 capable of performing:

- Battery Monitoring;
- Monitoring of poluents;
- Monitoring future and current Trouble Codes.

It is also worth mentioning that platform for customer feedback in the context of Industry 4.0 is still a great challenge and are points that deserve appropriate studies in the academic area [12,50].

## 3. Platform Architecture

**Figure 2:**
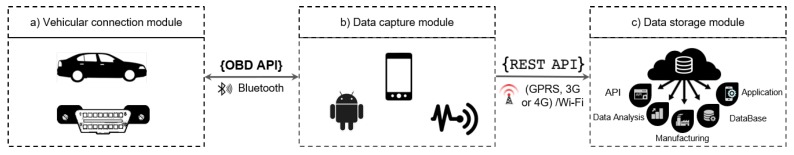
Architecture Overview.

The main objective of the proposed platform is to provide a feedback structure in order to communicate the vehicle’s data to the manufacturers. An overview of architecture is described in Figure 2 where is possible to highlight three main modules as follows:

(a) Vehicular connection module: which aims to connect the vehicle ECU to the other platform devices through an off-the-shelf OBD-II device;
(b) Data capture module: whose objective is to be a gateway between the vehicle and the cloud server, communicating all data from any support communication protocols;
(c) Data storage module: is responsible for storing and data analytics from a Representational State Transfer (REST) API.

### 3.1. Vehicular Connection Module

**Figure 3:**
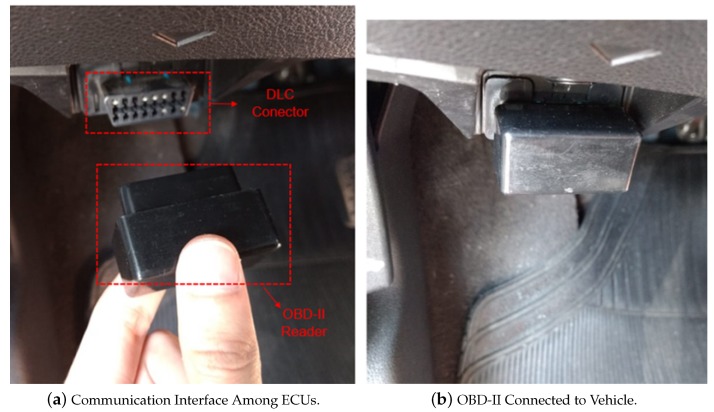
Vehicular Connection.

The vehicular connection is performed by means of OBD-II technology, which provides a real time communication interface among ECUs, sensors and actuators present in the vehicle, according to Figure 3a,b. The interface connector of OBD-II, known as the Data Link Connector (DLC), is supported in practically all vehicles produced since 1996 in US, 2003 in EUR, and 2010 in Brazil, China and Russia. A typical off-the-shelf OBD-II device (also referred to as “OBD scanner”) is able to connect to a DLC and act as a bridge between external applications (using Bluetooth or Wi-Fi protocols) and the vehicle (using most commonly communication protocols, such as: Controller Area Network (CAN), SAE J1850 PWM, SAE J1850 VPW, ISO 9141-2 and ISO 14230 KWP2000) [51].

Additionally, OBD-II technology supports 10 operating modes. Each of them has a specific series of commands which returns data from sensors and actuators present in the vehicle. To request such data, codes called Parameter Identification (PID) are used. However, automakers are not obliged to support all modes of operation in their vehicles. Thus, it is necessary to discover a procedure in order to decode all PIDs supported by a vehicle. Table 2 synthesizes the respective modes of operation.

### 3.2. Data Capture Module

**Figure 4:**
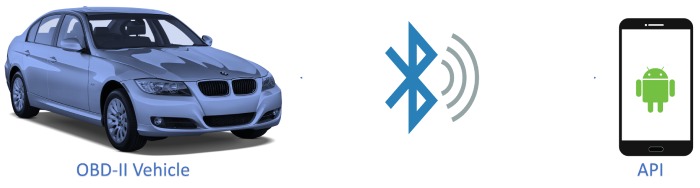
Connection to the vehicle and an Android Smartphone.

This module aims to capture the data from the vehicle through the use of a mobile application and send them to a database in the cloud. For this purpose, an API was developed to make the connection between the OBD-II device connected to the vehicle and an Android smartphone, according to Figure 4. The information capture procedure, by default, is performed every second, but this behaviour is configurable by the user.

The API was developed based on the Java language, intending to be used as a library in a holistic way for any typical Android application. The API is based on the OBD-II Java API made available by Paulo Pires on the GitHub code-sharing website (https://github.com/pires/obd-java-api) and which had its development suspended in the year 2017. During the development of this new library, all repositories created from the previous Paulo Pires’ API were analyzed (by means the of *forks* from *GitHub*), merging all changes, corrections and improvements. In that way, the new API was also made available on Github (https://github.com/eltonvs/java-obd-api) and it supports the entire strip of commands mentioned in Table 2.

The design pattern *composite* was used to allow the aggregation of commands in API. Thus, an application can create a group of commands to be executed in the OBD-II device. This procedure substantially improved the performance of the previous API developed by Paulo Pires. The execution for each group of commands can occur sequentially or in a concurrent way, being defined by the signature of the run method implemented in library.

The Fog Computing paradigm was also used in the API. It defines the architecture that extends the computational capacity and storage of the cloud to the access layers of the network, allowing the data to be analyzed and transformed into information or actions before being implemented [52]. Thus, it used the paradigm with the processing and decoding of the answers obtained by the vehicle. Because the CAN protocol operates synchronously, the API was developed to support concurrency, ensuring synchronization in the execution of the commands and allowing operations to occur concurrently, decreasing latency in execution and processing of commands. For this, locks were used as a synchronization method.

**Figure 5:**
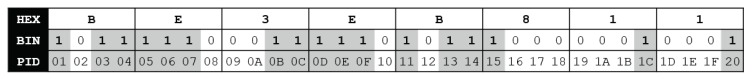
Decoding of the Supported PID Command.

Another important issue addressed by the API was the discovery procedure used to identify the commands supported by the vehicle. That algorithm is performed from the command “01 00” (mode 01, PID 00). The result (returned in hexadecimal base) has four bytes and is converted to an unsigned 32 bits integer. The PIDs supported by the vehicle can be expressed by the equations:

(1) S = {p ∈ N|1 ≤ p ≤ 32 ∧ bitseq(p) = 1}

(2) bitseq(n) = number ≫ (32 - n) & 1

where *S* is the set of PIDs supported by the vehicle whereas bitseq(p) is the function that convert the result coming from vehicle for an adequate representation. That function perform (32-n) displacements to the right and after that, a logical operation of and with 1, thus returning the value of the *n*th bit of the number. That algorithm can be better demonstrated in Figure 5 (where the darker columns represent the supported commands).

Also related to supported commands by API, an agnostic implementation of the data flow based on InputStream and OutputStream Java classes was developed for the request and response of the commands. Thus, the application is not limited only to a communication protocol (like CAN, SAE J1850 PWM, so on) or specific OBD-II devices.

**Figure 6:**
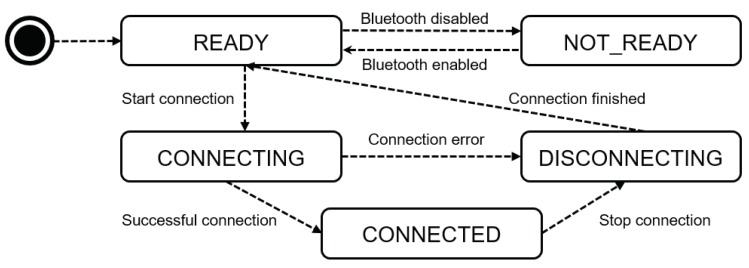
States Machine Orchestrations for the API.

Regarding the prediction of errors and energy consumption issues, a state machine was implemented in the mobile application. All states are described in Figure 6 and each of them has the following features:

(a) Ready: represent the initial state of the application and only from it can actions of the system be executed;
(b) Not Ready: state when the application requirements are not attended, blocking the application until they are met;
(c) Connecting: represents the state when the connection is started. Here, the connection to the OBD-II interface is established and a communication socket is created. If an error occurs during this process, the state changes to “*disconnecting*”.
(d) Connected: is reached if the connection was established correctly in the previous state, it is where the data exchange occurs through the previously created socket;
(e) Disconnecting: is the state used to terminate the connection with the vehicle. It can be reached through a user action (disconnect) or in the case of an error occurrence during connection. In this state, the cleaning actions and the closing of the connection take place, returning to the initial state of the application.

A central point handled by API is to decide what communication technology will be used to send data to the cloud server. A smart communication module was implemented to decide if GPRS/3G/4G or Wi-Fi technologies will be used depending on availability at the moment and user preferences (there is the possibility of disabling the mobile data usage in the application). Wi-Fi connections have a higher priority and are preferred when they are available.

Finally, the data collected by the application is sent to the cloud storage server using an optimized format for web applications based on JSON (JavaScript Object Notation) [53]. An example of supported format follow as:

{
    "dateTime": "2018-01-01 00:00:00",
    "data": [{
	"serial": "93YBSR7RHEJ2*****"",
        "GEOLOCATION": "{			  
		  \"altitude\": 45.835,
		  \"latitude\": -5.8321709,
		  \"longitude\": -35.2076702
	}"
        "AIR_INTAKE_TEMP": 53.0,
	"DISTANCE_TRAVELED_AFTER_CC": 63847,
	"DISTANCE_TRAVELED_MIL_ON": 0,
	"DTC_NUMBER": 0,
	"ENGINE_COOLANT_TEMP": 91.0,
	"ENGINE_LOAD": 47.058823,
	"ENGINE_RPM": 786,
	"INTAKE_MANIFOLD_PRESSURE": 46,
	"LONG_TERM_BANK_1": -3.90625,
	"PENDING_TROUBLE_CODES": "[]",
	"SHORT_TERM_BANK_1": -1.5625,
	"SPEED": 17,
	"THROTTLE_POS": 16.078432,
	"TIMING_ADVANCE": -28.5,
	"TROUBLE_CODES": "[]"
 }]
}


As explained in the previous example, the JSON has 2 fields. The former, named dateTime, corresponds to the time-stamp when the data collection was performed. The latter, named data, holds all information about the vehicle, including its serial number (filled using the Vehicle Identification Number (VIN)), geo-location, and all sensor data.

### 3.3. Data Storage Module

This platform module is responsible for storing and distributing the data and can be extended by the creation of submodules. Its main submodule is the “Vehicle Data Historian”, providing an API (used by the mobile application for communication with this module) in which any sub-application can insert or access the data stored in the history through its access points. The idea behind the decentralized development of this module is to allow the expansion and development of the sub-applications following the principle of single responsibility. This enables the creation of specialized submodules in each part of the platform’s data analysis, processing and visualization. In addition, from that same submodule, it is possible to support the process of smart manufacturing vehicles, providing insights, analysis and detection of defective parts in the production line.

In summary, the operations supported by this module are as follows:

(a) Registration: in this operation the vehicle registration is preformed, informing the model name, serial number and supported sensors;
(b) Identification: the identification of the attribute types (number, Boolean, point, string, among others) which the available sensors work with. In other words, the structure of the JSON used to send the data is defined with all the necessary data types;
(c) Verification: the process of verification both of the registered vehicle and the type of data that is sent;
(d) Storage: Sends and receives information through a REST API making use of standard HTTP methods. The data coming from the vehicle sensors is structured in JSON format as cited in Section 3.2. This data is then stored in a database and made available for reading as a time series.

As for the interactions with the Storage module, a REST API is used, since the devices prepared for IIoT and/or Industry 4.0, even with the variety of standards and protocols, can send and receive data via APIs in the REST standard. As it is an open, format agnostic, lightweight, asynchronous, stateless and globally accepted standard, which makes integration with other API systems simpler [54].

To accomplish these tasks, the module uses the concept of containers. This technology enables the creation of multiple isolated instances of a given operating system inside a single host, i.e., provides the means to virtualize applications inside a server [55,56]. Since new instances of the application can be created when the system is under heavy load, it is possible to accomplish horizontal scalability and data elasticity. As a result, allowing the service to remain stable and working properly even when there is a large number of simultaneous requests [56].

**Figure 7:**
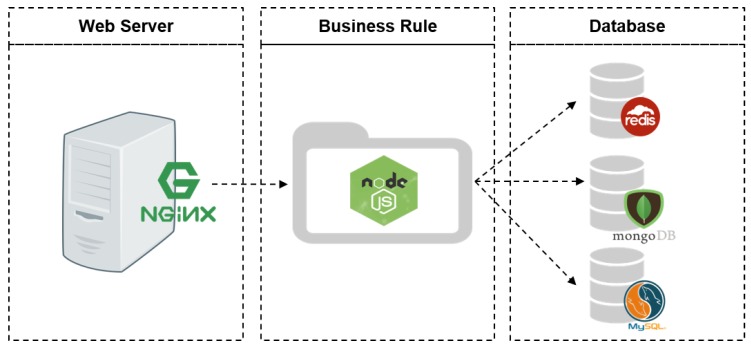
Structure of the Data Storage Module.

The environment is composed of three layers: Web Server, Business Rules and Database, as illustrated by Figure 7.

The Web Server layer makes use of the NGINX (https://www.nginx.com/) tool, which is intended to act as a reverse proxy, providing static files with a better performance. The Business Rule layer uses Node.js (https://nodejs.org/en/), which is a framework suited for development of high performance applications that deal with asynchronous flow for reading and writing data (i/o). This behaviour allows multiple connections to be maintained and processed simultaneously by the Node.js, which receives the requests, analyzes what is being requested and stores what is received. The third and last layer is the database, which manages the reading and writing of data and automobile management (registration an editing). This process is made in three distinct databases to be consumed by the application that runs on Node.js on the layer above.

In this context, it is known that data storage is of utmost importance. In order to store the data safely, it is necessary to know its type and processing priority. Therefore, three different databases are used for distinct processing purposes and access needs: Redis, MongoDB an MySQL. Each of these solutions have advantages and disadvantages as regards to performance [57].

The first one, Redis (https://redis.io/), is a non-relational database that stores the data in memory and has a high read-write performance compared to the Disk Management Database Management System (DBMS). In our application, Redis is used to store the data that needs to be accessed quickly, for example, the verification of the sensors.

MongoDB (https://www.mongodb.com/) is also a non-relational database, but, contrary to Redis, it uses disk storage. Even so it is an excellent solution for storing large amounts of data, such as the records collected by the vehicle.

Finally, there is MySQL (https://www.mysql.com/). Unlike the two previously mentioned databases, MySQL is a relational DBMS that performs well both on data indexing [57] and on disk storage. This behaviour ensures consistency between table relationships and, for this reason, is the preferred solution to store the vehicle registration data.

## 4. Evaluation

This section describes an evaluation of our approach, with will focus on the experiment definition and planning. The following section presents the obtained results.

### 4.1. Goal Definition

The main goal of our study is to investigate the customer feedback platform for Intelligent Vehicles in an Industry 4.0 perspective. Analyzing the use of the platform with the purpose of evaluation with respect to customer feedback of the vehicles in the context of drivers in Italy and Brazil.

### 4.2. Planning

This subsection details all evaluation design.

#### 4.2.1. Context Selection

The evaluation will target vehicles manufactured in Brazil since 2010 and in Italy since 2003, As these were the years in which the implementation of the OBD-II system on vehicles began to be mandatory in each of the countries.

#### 4.2.2. Research Questions

The issues we are trying to explore are as follows:

(a) Question 1: which are the sensors supported by each vehicle?
(b) Question 2: what are the speed patterns used by the drivers in urban routes and in highways?
(c) Question 3: what is the correlation of Speed to Revolutions Per Minute (RPM)?
(d) Question 4: what is the battery behaviour when the vehicle is in operation?
(e) Question 5: what are the error codes presented in each of the vehicles?

#### 4.2.3. Selected Sample

The sample selected was based on the convenience and availability of the drivers, they were distinct vehicles in two countries, as described in the (Table 3).

#### 4.2.4. Instrumentation

The instrumentation process will be done initially with the configuration of the environment for the experiment and planning of data collection procedure. This will be held with the installation of APP in the smartphone.

Here are the resources to use.

**Figure 8:**
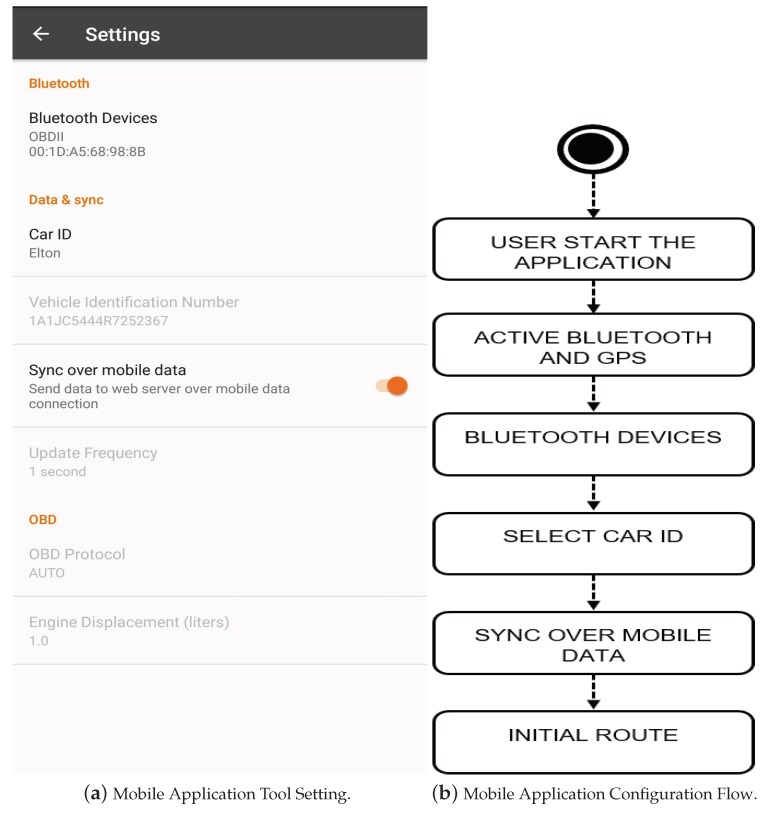
Mobile Application.

(a) Connecting the OBD-II: in the vehicle, later on, the smartphone to OBD-II. it was in Section 3.2.
(b) Mobile Application tool setting with OBD-II: is shown in the Figure 8a,b. You only need to configure Bluetooth Devices by selecting the OBD-II that is connected to your smartphone, define o Car ID, the other settings are already set by default.
(c) Initial route: in different roads (highways and urban perimeter).

#### 4.2.5. Execution

Data was collected for around 1000 km. From those, 25% were collected on highways and 75% on urban routes.

## 5. Results and Discussion

This section addresses the results obtained in the evaluation. With relation to the research questions previously proposed (Section 4.2.2).

### 5.1. Research Question 1

The first analysis was to evaluate the practical feasibility regarding the capture of the vehicular sensors (Question 1). It is known that the OBD-II has access to six groups of variables (Control, Motor, Fuel, Pressure, Temperature and Others) that aims to guarantee the analysis of vehicle components (sensors), but not all vehicles have all sensors supported. Thus, for vehicles of the sample cited in Section 4.2.3, the results of the number of available sensors are shown in Table 4.

In Table 4, it can been observed that some vehicles have more sensors than others. Some factors that may have influence over this trend are the year of fabrication as well as the number of accessories in the vehicle itself. Thus, the greater the number of sensors, the greater and better the investigations with respect to the vehicle can be made. In this sample, both the Hyundai HB20 and Nissan Kicks stand out, being the newer and more complete cars of the group, they come equipped with a greater number of sensors (37 and 38 respectively). Among those, can be highlighted sensors that monitor battery voltage, ambient temperature, Fuel Level, and many others.

After the verification of the practical feasibility of the sensors available on the vehicle sample, we can start to answer the remaining questions. As we have access to detailed temporal data from each sensor in all of the vehicles, it is possible to correlate important data and compare the behavior of the vehicles under different operation conditions.

### 5.2. Research Question 2

For Question 2, in which we asked what are the speed patterns used by the drivers in urban routes and in highways. As it is known, there are several factors that limit the possible speed in every type of route, like safety and traffic regulations for example, as well as that excess speed may cause a greater fuel consumption.

**Figure 9:**
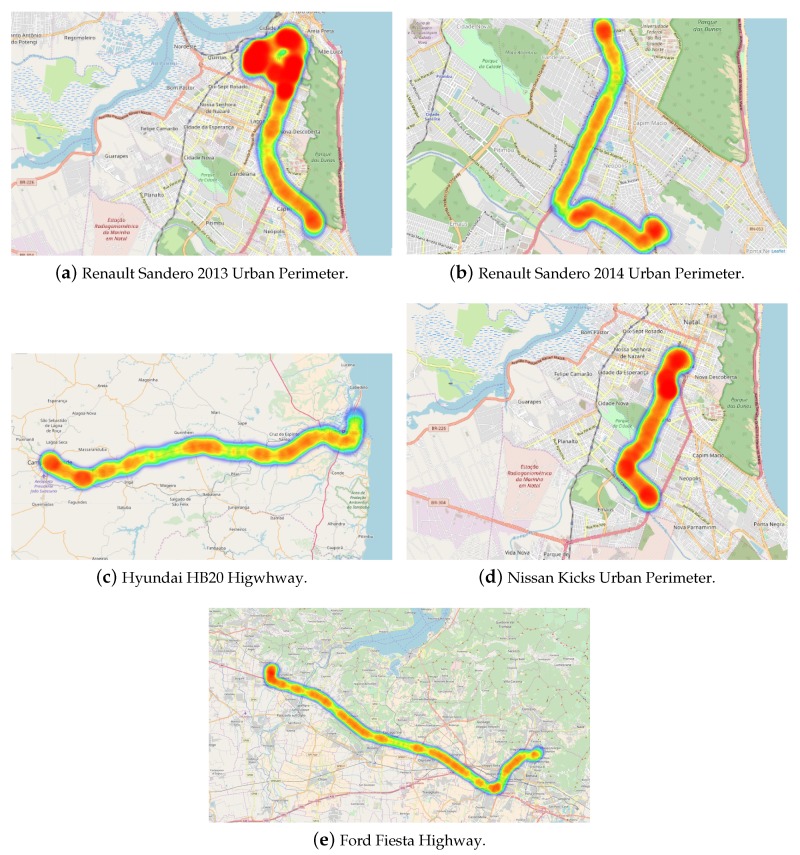
Heatmap of Speed en Route (Highway or Urban Perimeter).

With this in mind, the latitude, longitude and speed measurements were used to plot heatmaps for routes performed on each of the available vehicles. The 2013 and 2014 Renault Sandero and the Nissan Kicks were observed in urban routes in Brazil, where the speed limit is mostly 60 km/h. As it can be seen on the heatmaps shown in Figure 9, the three cars (a, b and d) reached the velocity limit several times during the routes. As a complementary investigation, the Hyundai HB20 and Ford Fiesta were used to analyze the speed patterns on highways, where the speed limits are considerably higher and there are fewer obstacles, like traffic and stop lights. This test was made both on Brazilian and Italian highways (HB20 and Fiesta respectively) and the results were consistent with the previous ones, as can be seen on Figure 9c,e.

**Figure 10:**
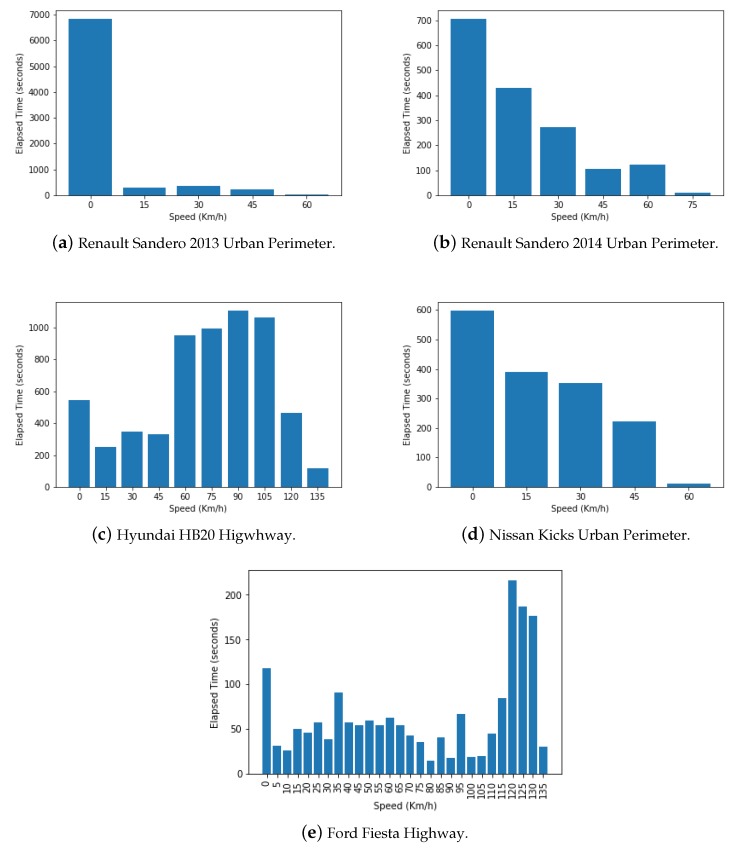
The Histograms of Speed on the Route.

In addition to the heatmaps discussed above, bar plots were used to help visualize how much time each of the vehicles spent traveling on various speeds, as can be seen in Figure 10. For the 2013 and 2014 Renault Sandero and the Nissan Kicks that, as mentioned previously, had their routes in urban centers, is clear to see that a greater portion of time was spent traveling at slower speeds and rarely near the speed limit (60 km/h), as shown on Figure 10a,b,d. This is probably due to the slower flow of traffic in urban areas, as well as a great number of stops due to traffic lights and traffic jams. As for the Hyundai HB20 and Ford Fiesta, which were tested on a Brazilian and an Italian highway respectively, have their results shown on Figure 10c,e. It is very clear to see that a greater period time was spent traveling at higher velocities and near the speed limit (between 80 and 100 km/h in Brazil and over 130 km/h in Italy), due to better traffic flow and higher speed limits than in urban areas.

### 5.3. Research Question 3

With relation question 3 we tried to verify what is the correlation of Speed to Revolutions Per Minute (RPM). First we need to understand the concept of RPM, which is the unit that measures the number of times the crankshaft of the engine rotates per minute and is closely related to the torque generated by the engine at any given time. In addition to that, the gearbox is composed of a series of gears and is responsible for transferring the engine power to the differential or to the wheels and helps maintain the RPM in a certain range. The gearbox transforms power into force or speed according to need. The higher the engine speed in relation to the axis the greater the force, and when the engine speed is smaller in relation to the axis, the higher the speed.

**Figure 11:**
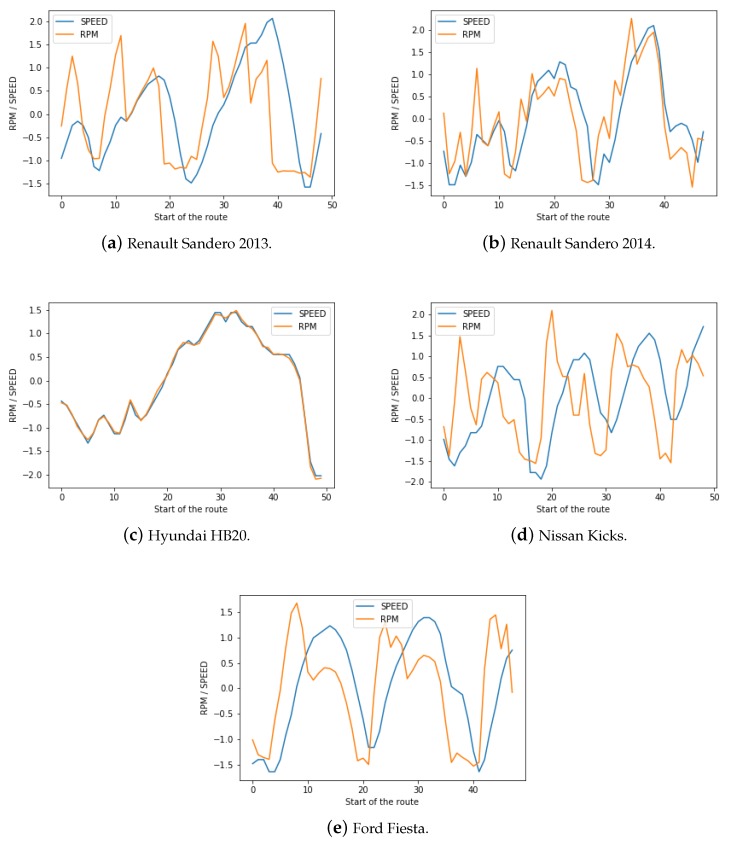
Race in RPM/Speed in Routes.

Since the RPM and Speed are variables with different order of magnitudes, the data obtained for each of the vehicles was normalized. This action enabled the visualization of both variables in the same graph and can be seen in Figure 11.

In Figure 11a,b, it is possible to see that the cars have a delayed response to the acceleration due to the small motor i.e., when the car is accelerated from the rest position, the RPM increases much faster than the speed, as much torque is need to break the inertia. It is also noticeable in some moments that the drivers do not need as much power as we see the RPM drop below the speed, which may indicate that the driver changed gears too early. Alternatively, we can also see the opposite behavior, where the driver lets the RPM go up too much before changing gears. In Figure 11c we can see a nearly ideal RPM/Speed relationship, where both of them vary together. However, unlike the measurements made for (a) and (b), this values were obtained on the highway route and after the car had already started. The vehicle on Figure 11d is different from the rest, as it is the only one with automatic transmission, that is, it has an automatic gearshift system. As a result, we can see a much more regular gearshift pattern, but it is still influenced by the acceleration. Finally, the car in Figure 11e, as the cars (a) and (b), has a small and less powerful motor, leading to a similar pattern. However,unlike the previously mentioned vehicles, the measurements were taken on a highway route, which leads to a faster rate or acceleration.

### 5.4. Research Question 4

Additionally, analyses were made to verify the battery behaviour of the vehicles during operation (Question 4). It is known that the battery function is to power the entire electrical system of the vehicle. Special emphasis should be given to the supply of power to the starter motor that allows the engine to start and to power the electrical components when the engine is not switched on. The life of the battery is directly linked to the electric balance, which is the ability of the alternator to generate the same amount of energy as the electrical system of the vehicle consumes. Thus, it can be said that the electric balance happens when, with the car in operation, the alternator can feed all electric components without relying on the battery. On the other hand, electrical imbalance occurs when the power consumed, on a given occasion, is greater than that generated by the alternator. As the energy consumed is not fully replenished, the battery voltage will decrease until exhausted if the energy consumed by the engine electronic management system and its components are not fully replenished.

**Figure 12:**
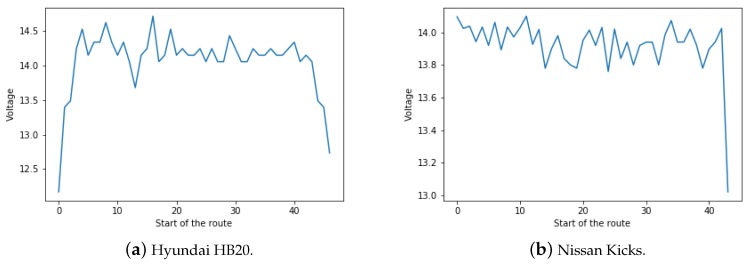
Representation of Data Collected by Voltage Control Module.

The electric balance is conserved when the voltage maintained by the regulator during the vehicle operation always vary between 13.8 V and 14.4 V. In contrast, when the car is turned off, the voltage should be around 12.4 V and 12.8 V. Thus, to answer Question 4, the battery voltage control module supported by the Hyundai HB20 and Nissan Kicks was analyzed to verify the battery behavior when the vehicle is in operation, as shown on Figure 12.

In Figure 12 it can be seen that the data collection process for the Hyundai HB20 (a) was started before the car was turned on, as demonstrated by the battery voltage being lower than 13 V at the beginning of the plot. However, the voltage was even lower than that of what is expected for the car turned off (12.4 V). As a result, the driver was contacted and informed that, since the car purchase (2015 Model), the battery has not been replaced. As for the Nissan Kicks (b), the data capture process was started after the car was turned on, so we see the battery voltage fluctuating between the expected levels. After the vehicle is turned off, it can be seen the voltage dropping, but still remaining above the minimum.

### 5.5. Research Question 5

Finally, to answer Question 5, we identified which errors were found in the vehicle systems during the evaluation. It should be noted that they are pre-defined by the automakers and stored in the electronic modules that allow the identification of faults in the vehicle systems.

The format of the vehicle diagnosis is standardized by ISO 15031-6. The document recommends that, for the fault code format, the codes should be grouped into four distinct groups: body (B0–B3), chassis (C0–C3), powertrain (P0–P3) e network communication (U0–U3). That being the case, the code messages should start with 2 characters identifying the group, followed by 3 numbers that describe the specific error that was detected. Therefore, it can be seen in Table 5 that all the Trouble Codes found in the vehicles follow this pattern. It is also noticed from Table 5 that the Renault Sandero 2013 presents the problem “Catalyst System Efficiency Below Threshold (P0420)” and both the Nissan Kicks and Ford Fiesta exhibit a “Rear Speed Sensor Malfunction (C0300)” error.

These codes provide the mechanic with information about the system and the conditions under which the issue was identified. However, they cannot provide a clear solution to the real cause of the problem as they only indicate where to look.

After these brief analyses, we can see that there is plenty of potential to encourage the feedback between the vehicles and industry, allowing manufacturers to identify in which type of route (urban or highway) the car has greater use, which parts exhibit the greatest wear or even manufacturing errors in one series, among many others. This corresponds to the previously defined concept of customer feedback platform.

### 5.6. Threats to Validity

The threats to validity for the present study were:

- Geo-location, conclusion validity: routes that go through areas with no GPRS / 3G or 4G coverage do not store their geo-location data (latitude and longitude) for these areas, i.e., the sensor data is stored locally and transmitted to the server once connections is reestablished, but the route cannot be identified due to the lacking geo-location data.
- Appropriate instrumentation, internal validity: vehicles were evaluated on different routes and times, since it was not intended to make any kind of comparison between them, just check the viability of each sensor.
- Representative population, external validity: The variety of vehicles composing the sample was significant for the research purpose, however, there are vehicle models that have not been evaluated.

## 6. Conclusions and Future Works

Industry 4.0 involves the use of advances in communication and information technology to increase the degree of automation and digitalization of production, manufacturing and industrial processes. Its purpose is to manage the entire value chain process, improving efficiency in the production process and generating quality products and services. This requires intelligent systems and sensors that inform the machines how they should work and how they will be involved in each stage of the manufacturing process.

The processes must be self-managed in a decentralized modular system. Intelligent embedded systems begin to work in tandem with the exchange of data and information, both directly and through the cloud on the Internet.

It is known that monitoring and feedback mechanisms are traditionally time-consuming. With Industry 4.0, concepts and methods applied to logistics and statistics will be generated and collected in an automated way. The company will know immediately if an adjustment is to be made and what adjustments will be required to respond more quickly to customer needs.

Therefore, this article presents the proposal and a evaluation of a customer feedback platform for Industry 4.0. The platform aims to collect and analyze data from all sensors available by in vehicles through the OBD-II hardware. It is hoped that the proposed API can bring benefits to research and development in the area.

Although it is important to realize that some difficulties were encountered during the development of the platform, such as the time required to request the commands supported by each vehicle. This problem was solved after the development of an algorithm that could decode the supported commands, which enabled the reduction of the request time from 8 s to 1.5 s. Another difficulty was met during the evaluation period of the application. Since it is not a controlled environment (as in a simulator), the connection is prone to errors, which must be treated. This problem was solved by carrying out a pilot study with two cars, mitigating the errors.

For the evaluation of the platform five questions were defined for investigation, all of which managed to be answered even with the reduced sample size. With the questions, we identified the number of sensors in each of the vehicles, where the Hyundai HB20 and Nissan Kicks stood out for having the largest number of sensors (37 and 38 respectively). Regarding the speed sensor in different routes, it was noticed that the drivers spend a good amount of time with speed 0, which depicts the time spent in traffic and stop lights. Furthermore, for RPM and speed relation, it has been realized that cars with small and low potency motors need more torque to start moving, as well as that even in the car with automatic transmission, with its self-regulating gearshift system, the acceleration still will influence the RPM and speed relation.

Still with regard to the questions, it was verified the battery behavior in both cars that had the necessary sensors, the Hyundai HB20 and Nissan Kicks. For the former it was identified that the battery voltage was below the expected limit when the car was turned off, possibly indicating that the battery should be replaced soon. For the later, it was verified that the battery was operating inside the expected range of voltages. Finally, we analyzed the Trouble Codes presented by each of the vehicles, which are codes that allow us to identify vehicle failures.

In conclusion, this work has the intention to broaden and diversify the set of existing works in the subject and to collaborate modestly to the advance of research in the context of Industry 4.0 with instant feedback from the vehicles.

As future works include the addition of the instant fuel consumption algorithm, enabling pollution levels to be measured [35]; the use of “Protocol Buffers” to send data to the server, since this technology enables savings in the use of mobile data by mobile applications; analyze the State of Charge (SOC) of the vehicle batteries [15]; the enlargement of the evaluation for validation of the proposed platform; and the use of Big Data and machine learning techniques to detect patterns from the collected data.

## Figures and Tables

**Table 1:** Summary of Related Works.

	Features	Observed Sensors	Data Storage	Industry 4.0	App
Works					
[37]		Gas Analyzer	Storing on a smartphone	No	No
[39]		Latitude, Longitude, Altitude, Vehicular Speed (VS), Engine Coolant Temperature, Engine RPM, Ignition Timing Advance, Intake Air Temperature, AP, Mass Air Flow Rate (MAF), Manifold Air Pressure (MAP), Engine Load and Grade	Stored directly on a computer that is inside the vehicle connected to the output of the OBD-II adapter	No	No
[35]		MAF, MAP, Absolute temperature (IAT) and Engine RPM	Storing on a smartphone	No	Vehicle Data Collector
[38]		Fuel Level (FL), MAF, Fuel System Status (FSS), Long Term Fuel Trim (LTFT), Short Term Fuel Trim (STFT), VS, Engine RPM and Aceleration Position (AP)	Storing in dataset online	No	EcoDrive
[40]		MAF, Air to Fuel Ratio (AFR) and VS	Storing in dataset online	No	No
[41]		does not detail in the article	Storing on a smartphone	No	Industrial Engine Diagnostic System
[42]		Engine Load, Engine coolant Temperature, Intake manifold absolute pressure, Engine RPM, VS, Intake air Temperature, MAF and Absolute throttle position	Storing in dataset online	No	Idrive
[43]		VS, AFR, Engine RPM and Trouble Codes	Storing in dataset online	No	On-Line Service
[44]		VS	Stored directly on a computer that is inside the vehicle connected to the output of the OBD-II adapter	No	Smart Tachograph
[45]		Control Module Voltage	Stored directly on a computer that is inside the vehicle connected to the output of the OBD-II adapter	No	No
[47]		Did not use OBD-II	Storing in dataset online	Yes	No
[48]		Did not use OBD-II	Did not use	Yes	No
[49]		Did not use OBD-II	Not suitable	Yes	No

**Table 2:** Modes of Operation supported by OBD-II.

Mode	Description
01	Return the real-time ECU data.
02	Request the ECU data corresponding to the last failure.
03	Display the error codes stored in the vehicle.
04	Clear the stored error codes.
05	Return the test results of O_2_ sensors present on the vehicle.
06	Return the test results related to non-continuous monitoring.
07	Return test results related to continuous monitoring.
08	Require the control of the on-board systems.
09	Get vehicle information.
10	Displays the error codes with permanent status.

**Table 3:** Selected Sample.

Model	Year	Motor	Transmission	Fluel	Country
Renault Sandero	2013	1.0	Manual	flexible	Brazil
Renault Sandero	2014	1.0	Manual	flexible	Brazil
Hyundai HB20	2015	1.0	Manual	flexible	Brazil
Nissan Kicks	2017	1.6	Automatic	flexible	Brazil
Ford Fiesta	2009	1.0	Manual	flexible	Italy

**Table 4:** Number of Available Sensors per Vehicle.

Model	Year	Sensors
Renault Sandero	2013	22
Renault Sandero	2014	22
Hyundai HB20	2015	37
Nissan Kicks	2017	38
Ford Fiesta	2009	22

**Table 5:** Trouble Codes.

Vehicle Model	Error	Description
Renault Sandero 2013	P0420	Catalyst System Efficiency Below Threshold (Bank 1)
Renault Sandero 2014	None	-
Hyundai HB20	None	-
Nissan Kicks	C0300	Rear Speed Sensor Malfunction
Ford Fiesta	C0300	Rear Speed Sensor Malfunction

## Abbreviations

The following abbreviations are used in this manuscript:

AFR	Fuel Ratio
AP	Acceleration Position
API	Application Programming Interface
CAN	Controller Area Network
CARB	California Air Resources Management Committee
CPS	Cyber-physical Systems
DLC	Data Link Connector
ECU	Engine Control Unit
EPA	Environmental Protection Agency
ERP	Enterprise Resource Planning
FL	Fuel Level
FSS	Fuel System Status
GPS	Global Positioning System
IIoT	Industrial Internet of Things
IoIV	Internet of Intelligent Vehicles
IoT	Internet of Things
JSON	JavaScript Object Notation
LTFT	Long Term Fuel Trim
MAF	Mass Air Flow Rate
MAP	Manifold Air Pressure
MES	Manufacturing Execution Systems
OBD-II	On-Board Diagnostics
PID	Parameter Identification
REST	Representational State Transfer
RPM	Revolutions Per Minute
SOC	State of Charge
SOTA	Software Updates Over The Air
STFT	Short Term Fuel Trim
VANETs	Vehicular Ad-hoc Networks
VIN	Vehicle Identification Number
VS	Vehicular Speed
WPANs	Wireless Personal Area Networks
